# Supplementary material for: Pan-Cancer Analysis of the Roles and Driving Forces of RAB42
Source: Biomolecules. 2022 Dec 26;13(1):43. doi: 10.3390/biom13010043 (PMC9855782; doi:10.3390/biom13010043)
Supplement: Supplementary file 1 [file biomolecules-13-00043-s001.zip › biomolecules-2029114-supplementary.pdf]

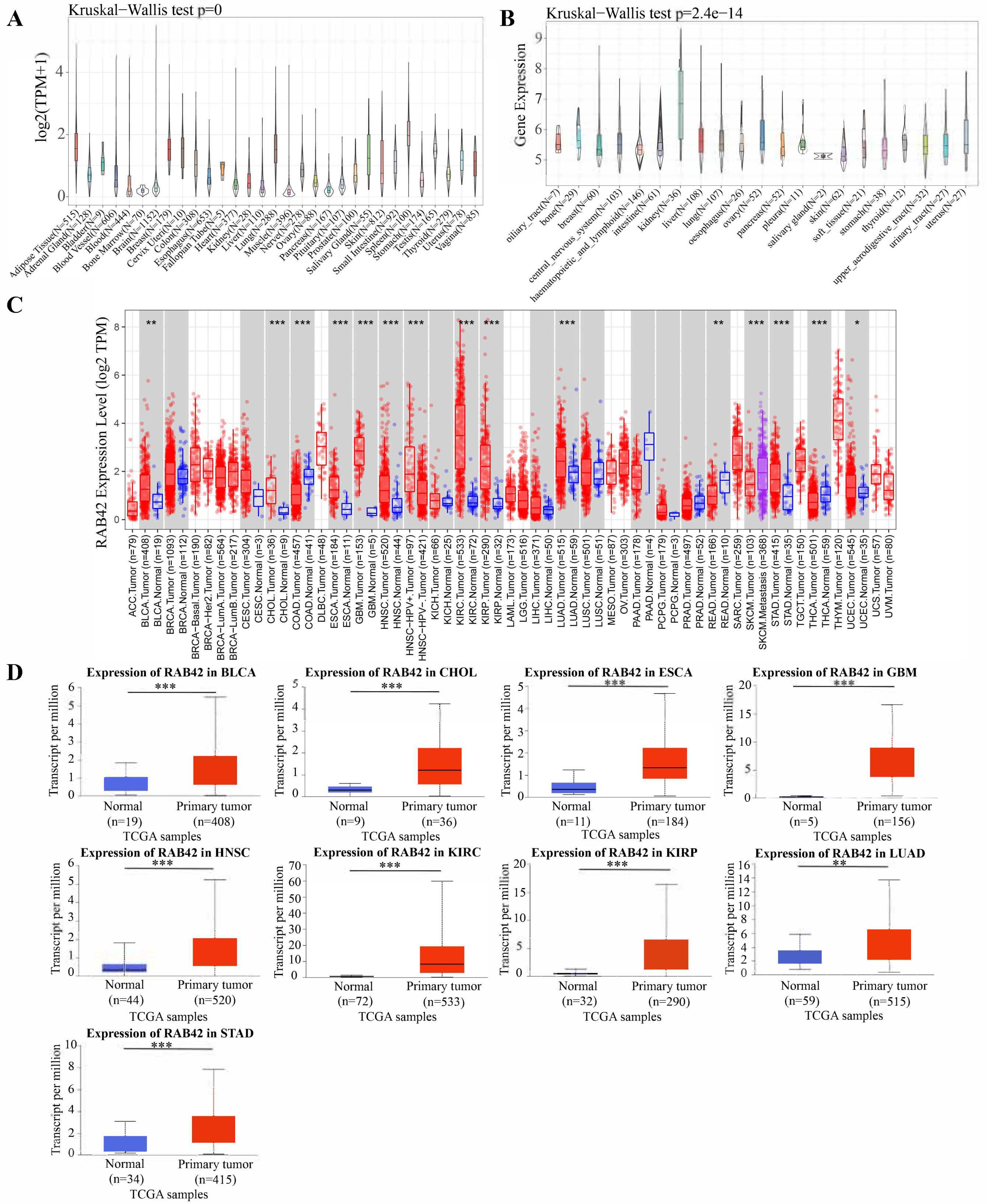

Supplementary Figure S1: Expression analysis of RAB42 in pan-cancer. The expression levels of RAB42 in normal tissues (A), cancer cell lines (B) using SangerBox database. (C) RAB42 expression in different cancers using TIMER2.0 database. (D) RAB42 expression in 9 tumor types using UALCAN database. *P < 0.05, **P < 0.01, ***P < 0.001.

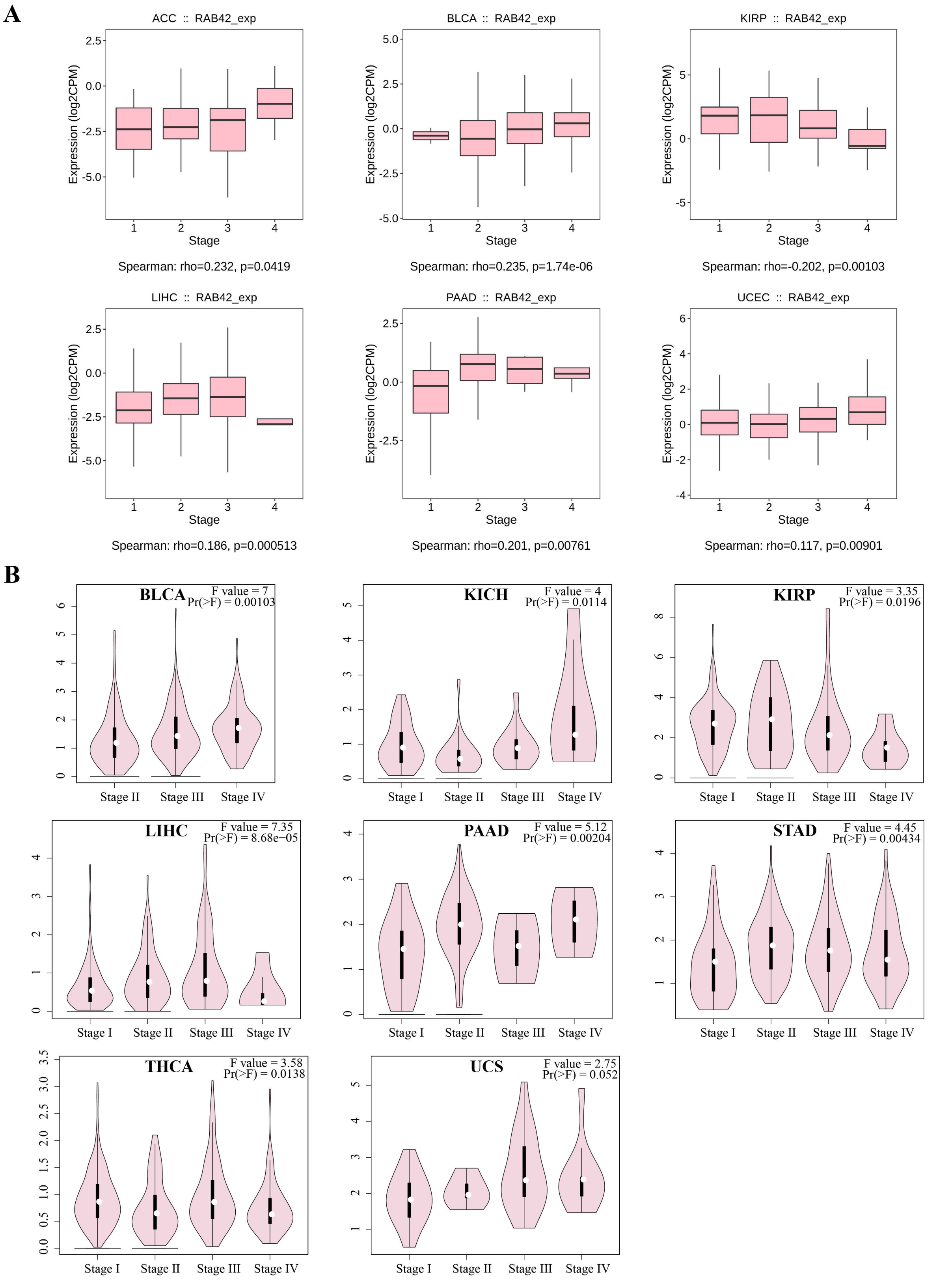

Figure S2: RAB42 expression in different pathological stages. RAB42 expression in different pathological stages using TISIDB database (A) and GEPIA2 database (B).

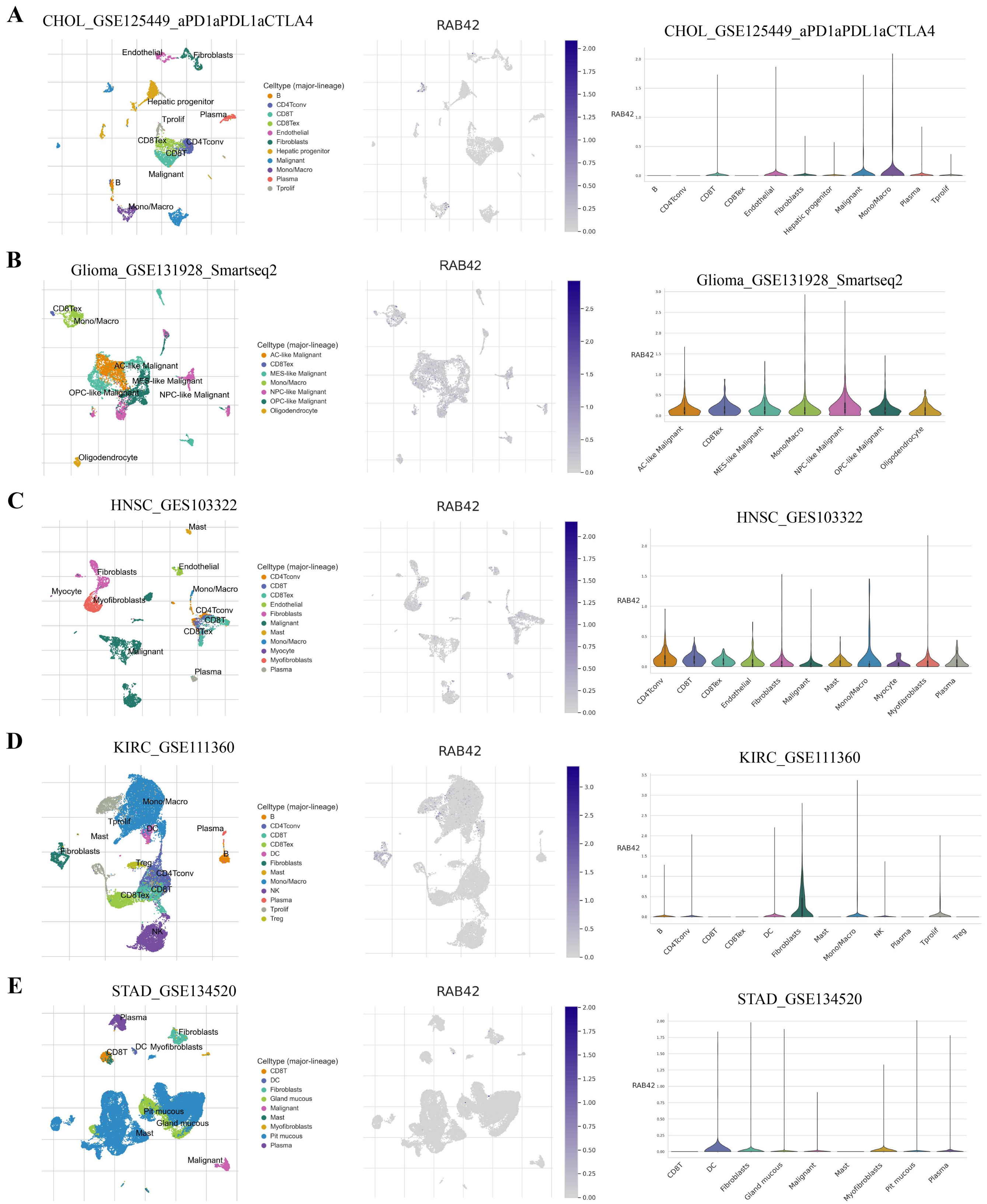

Figure S3: Single-cell sequencing analysis in different cancers. The expression of RAB42 in CHOL (A), Glioma (B), HNSC (C), KIRC (D) and STAD (E).

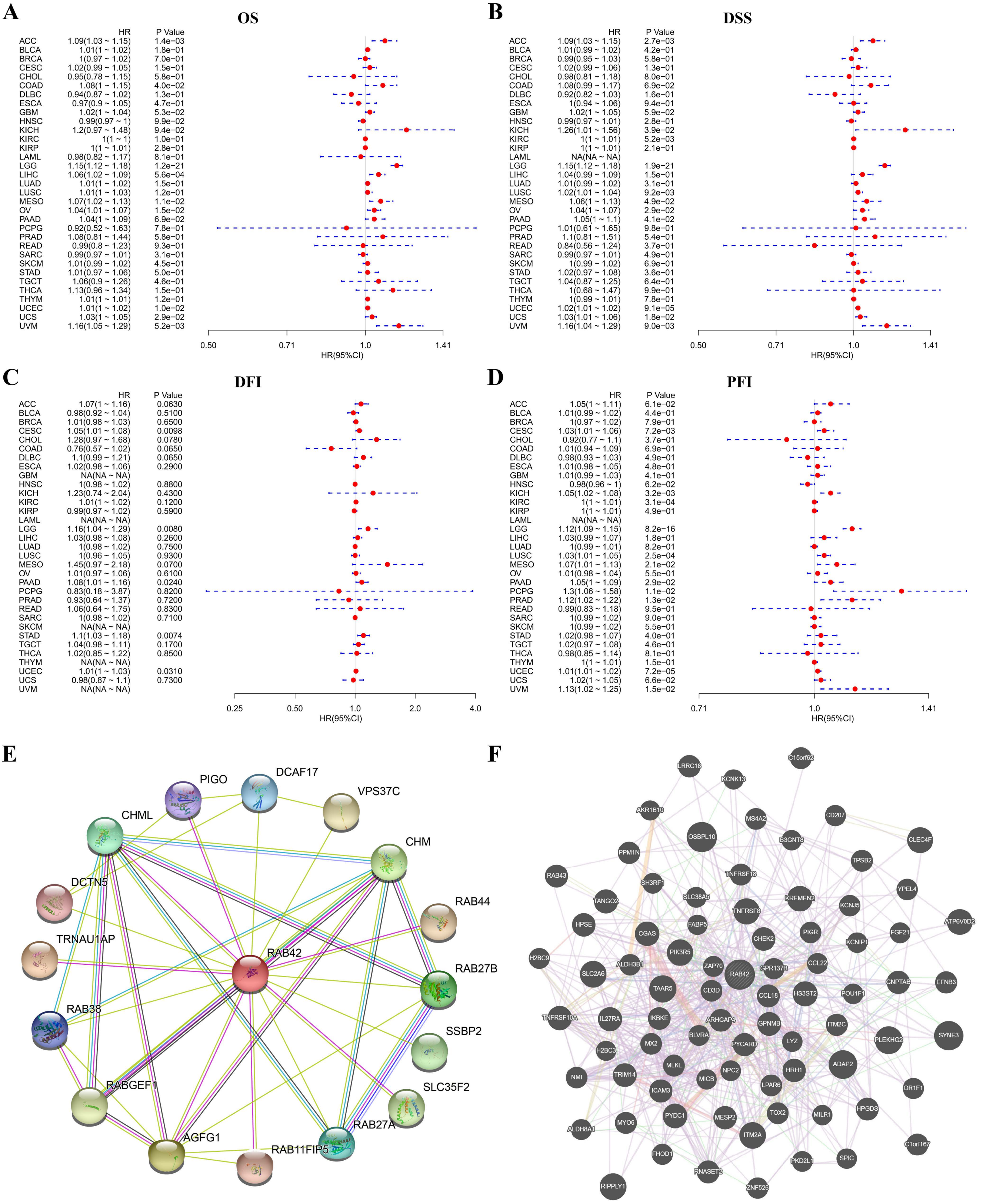

Figure S4: Forest plots of OS (A), DSS (B), DFI (C) and PFI (D). (E) The PPI networks of RAB42 using STRING online tool. (F) The interaction networks of RAB42 using GeneMANIA database.

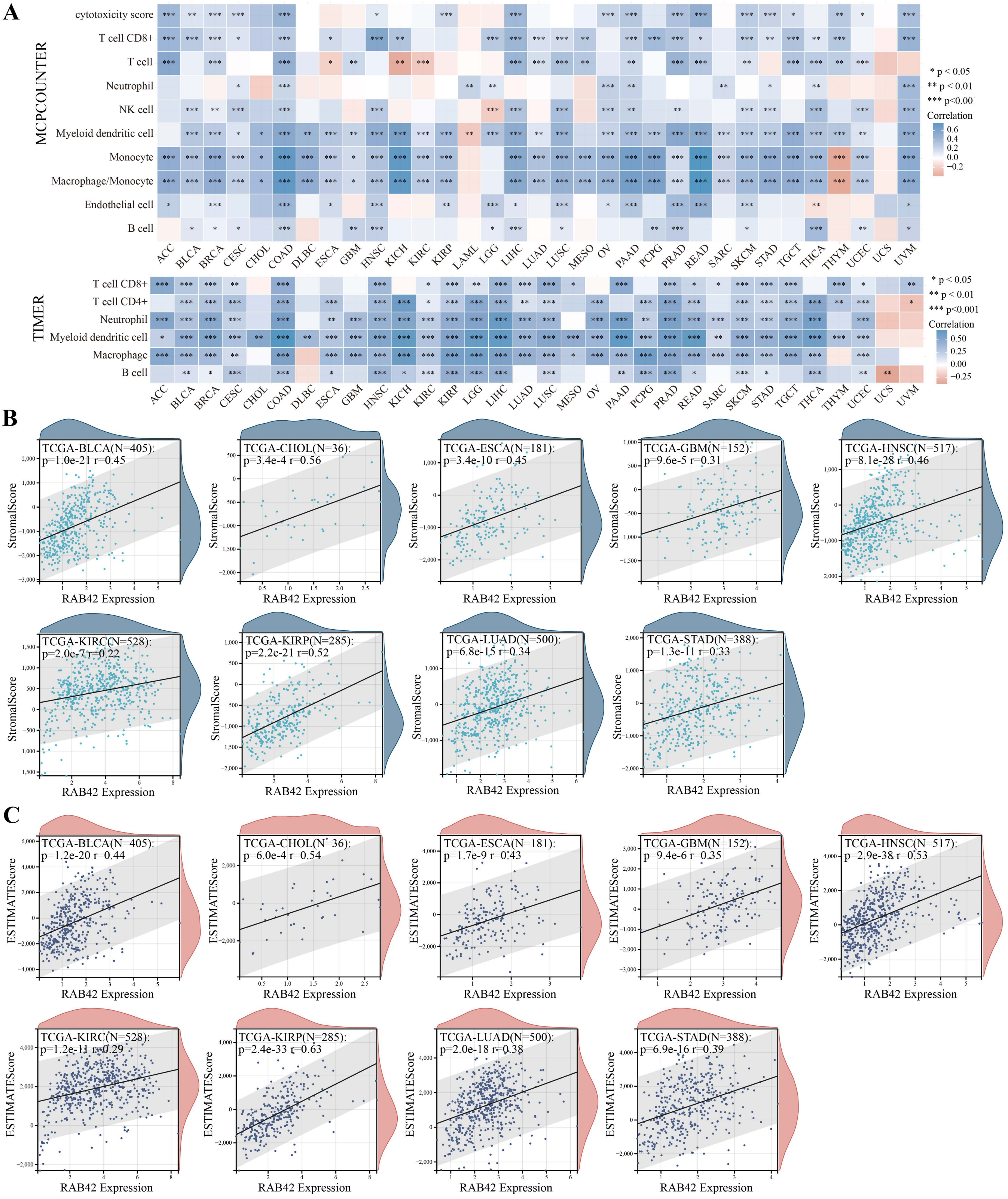

Figure S5: Correlation analysis between RAB42 expression and immune cell infiltration. (A) Spearman correlation between RAB42 expression and immune cell infiltration in pan-cancer. (MCPCOUNTER and TIMER algorithm) (B) Spearman correlation between RAB42 expression and stromal score. (C) Spearman correlation between RAB42 expression and ESTMATE score. *P < 0.05, **P < 0.01, ***P < 0.001.

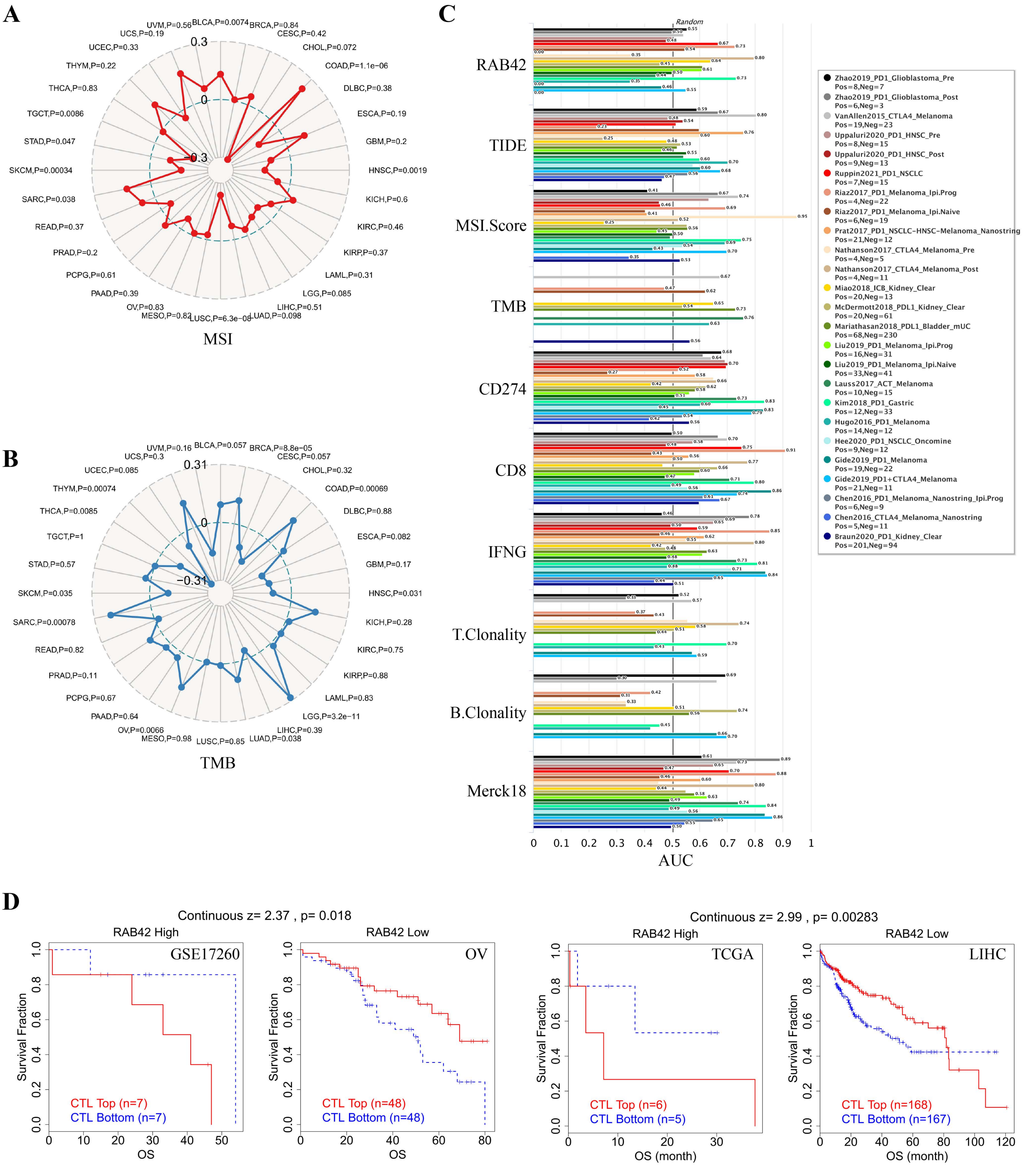

Figure S6: The predictive value of RAB42 for immunotherapy. (A) The correlation between RAB42 expression and MSI. (B) The correlation between RAB42 expression and TMB. (C) The predictive value of biomarkers for immunotherapy. (D) The overall survival of tumor patients with different RAB42 expression and CTL levels.

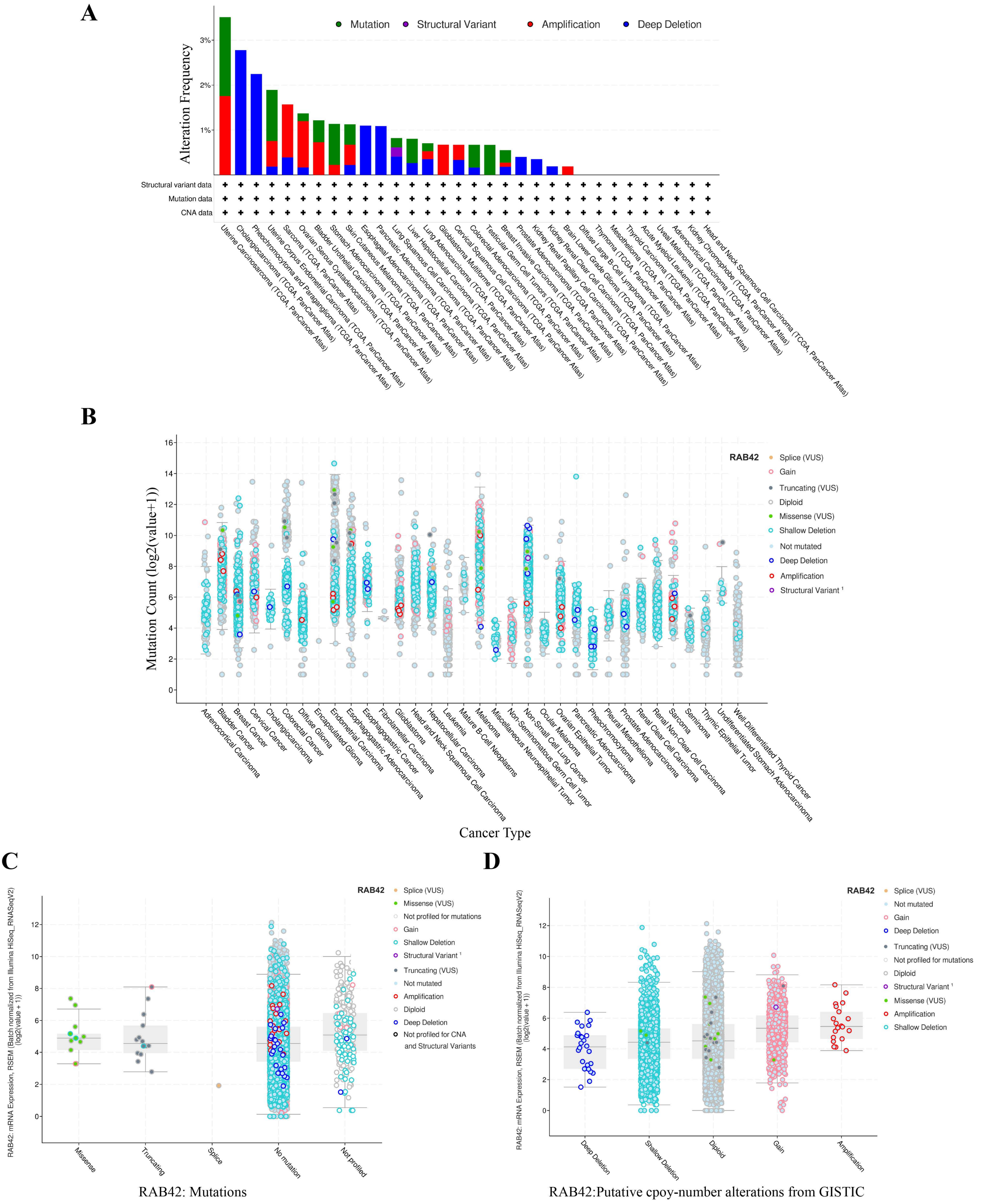

Figure S7: The mutation status analysis for RAB42. (A) The alteration frequency of RAB42 in pan-cancer. (B-D) The mutation counts, mutant types and copy-number alterations of RAB42.

A

Number	Region	Obs/Exp	%GC
1	464 to 663	0.71	50.50
2	465 to 664	0.70	51.00
3	466 to 665	0.68	51.50
4	467 to 666	0.68	51.50
5	468 to 667	0.67	52.00
6	469 to 668	0.66	52.50
7	470 to 669	0.66	52.50
8	471 to 670	0.64	53.00
9	472 to 671	0.63	53.50
10	473 to 672	0.63	53.50
11	474 to 673	0.62	54.00
12	475 to 674	0.62	54.00
13	476 to 675	0.62	54.00
14	477 to 676	0.68	54.50
15	478 to 677	0.69	54.00
16	479 to 678	0.69	54.00
17	480 to 679	0.62	54.00
18	481 to 680	0.64	53.50
19	482 to 681	0.64	53.50
20	483 to 682	0.63	54.00
21	484 to 683	0.64	53.50
22	485 to 684	0.63	54.00
23	486 to 685	0.64	53.50
24	487 to 686	0.63	54.00

B

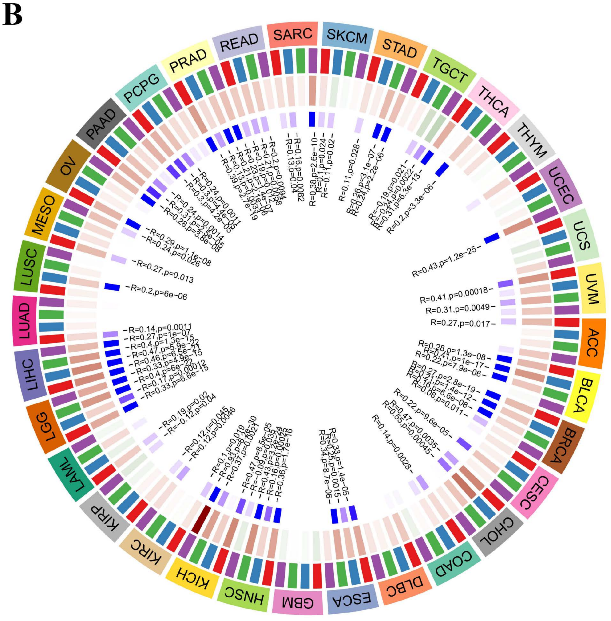

C

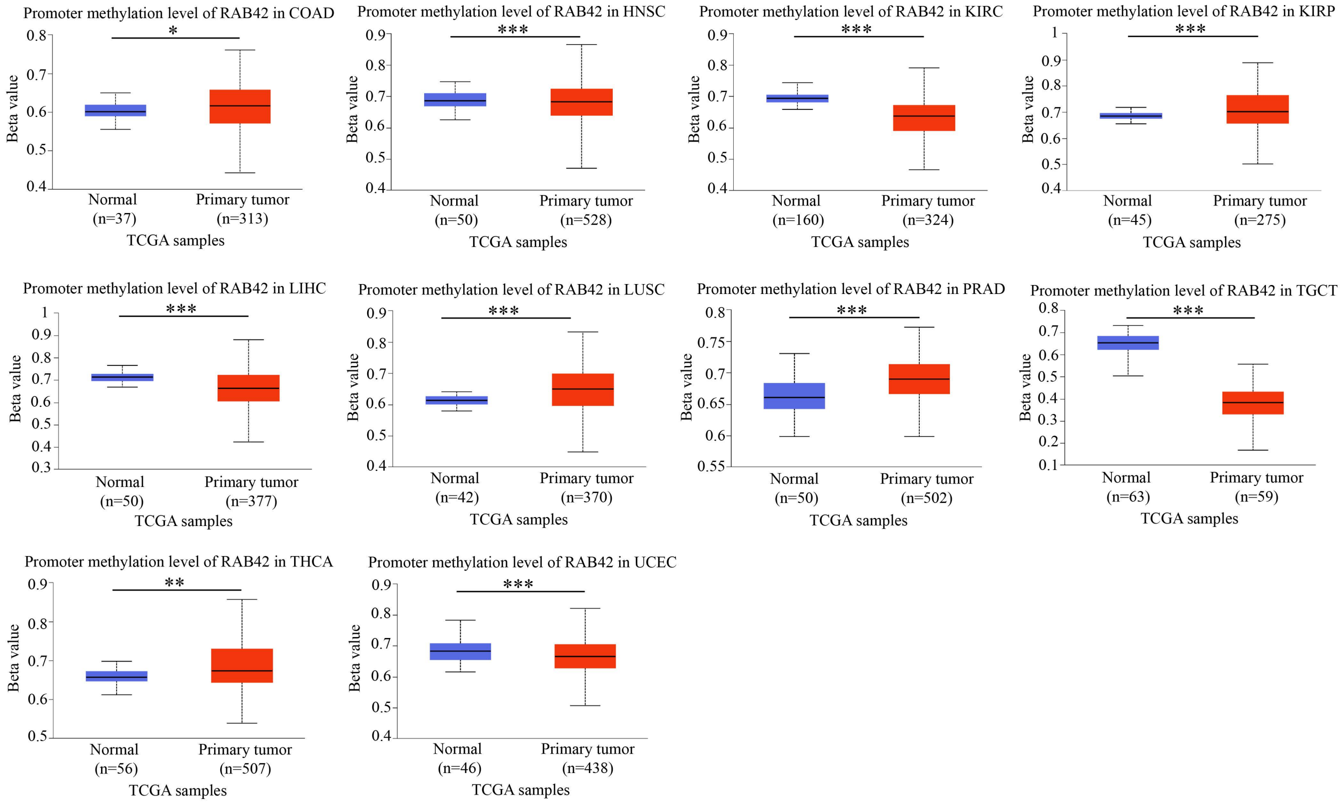

Figure S8: The correlation between DNA methylation and RAB42 expression. (A) CpG islands in RAB42 promoter region. (B) The correlation between RAB42 expression and DNA methyltransferases.Red box: DNMT1; blue box: DNMT2; green box: DNMT3A; purple box: DNMT3B. (C) Promoter methylation levels of RAB42. *P < 0.05, **P < 0.01, ***P < 0.001.

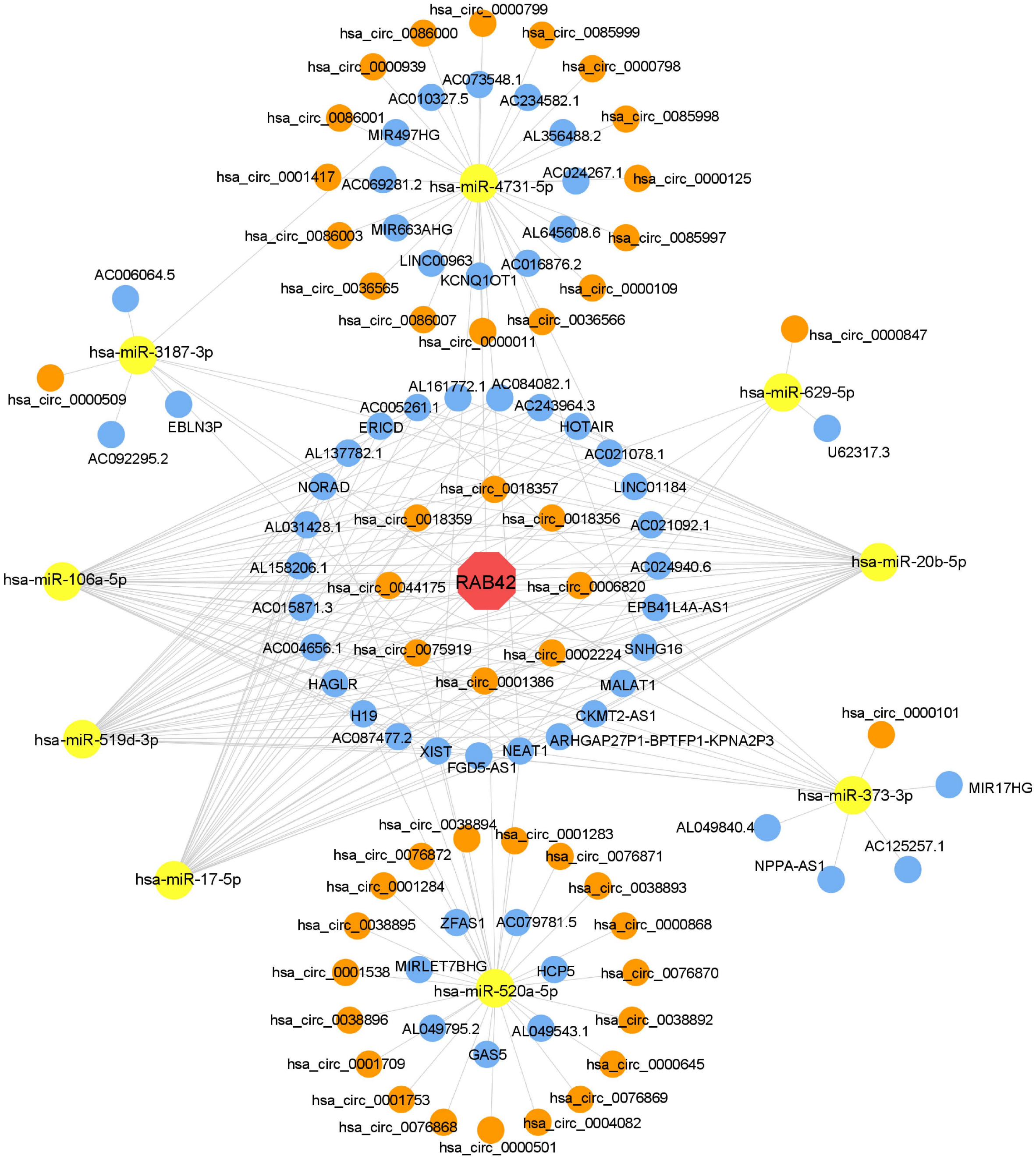

Figure S9: CeRNA regulatory networks for RAB42. Yellow circles represent miRNAs, orange circles represent circRNA, blue circles represent lncRNA.

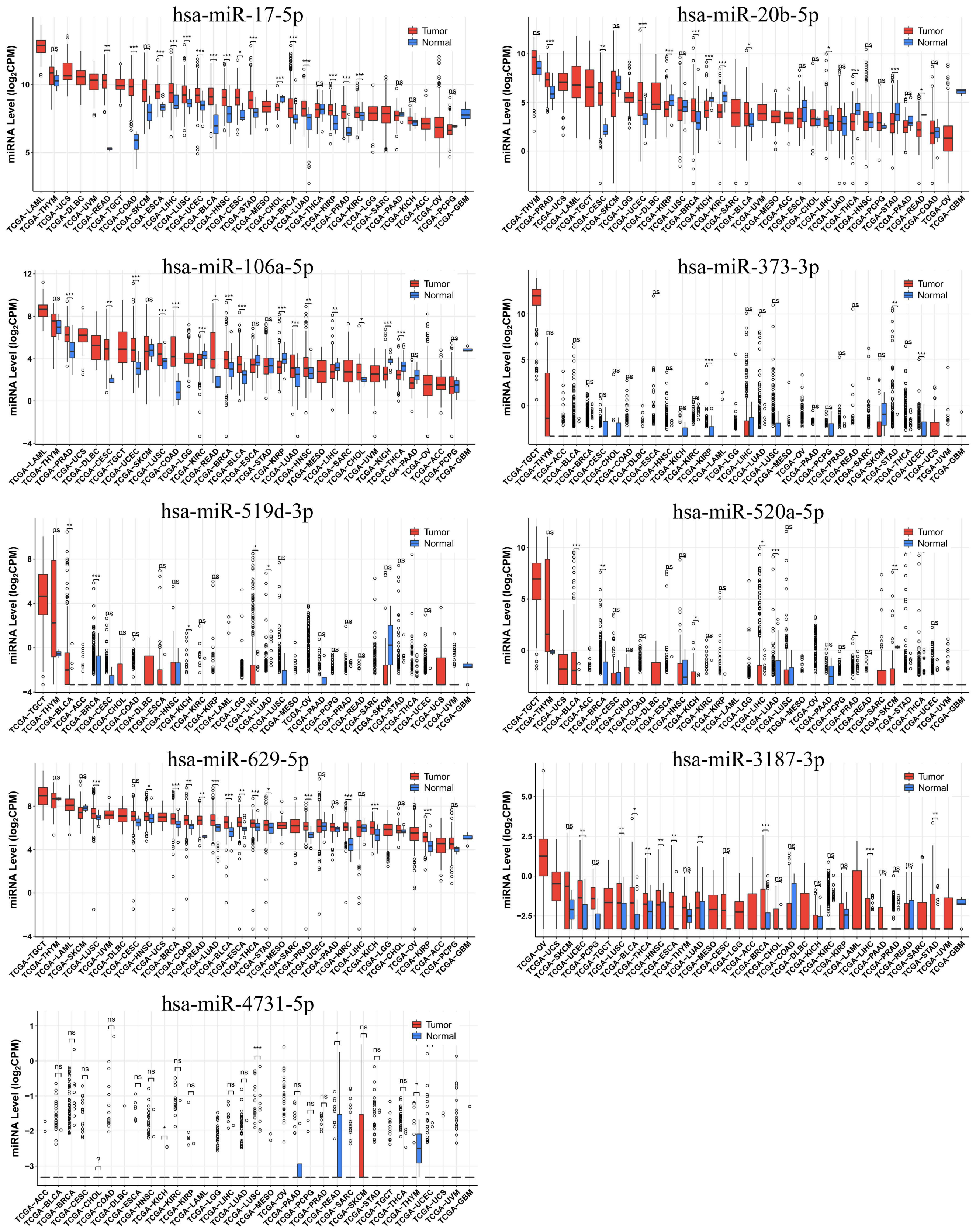

Figure S10: The expression of 9 miRNAs in pan-cancer.

TABLE S1 List of abbreviations.

Abbreviations	Full name
ACC	Adrenocortical carcinoma
BLCA	Bladder Urothelial Carcinoma
BRCA	Breast invasive carcinoma
CESC	Cervical squamous cell carcinoma and endocervical adenocarcinoma
CHOL	Cholangiocarcinoma
COAD	Colon adenocarcinoma
COADREAD	Colon adenocarcinoma/Rectum adenocarcinoma Esophageal carcinoma
DLBC	Lymphoid Neoplasm Diffuse Large B-cell Lymphoma
ESCA	Esophageal carcinoma
GBM	Glioblastoma multiforme
GBMLGG	Glioma
HNSC	Head and Neck squamous cell carcinoma
KICH	Kidney Chromophobe
KIPAN	Pan-kidney cohort (KICH+KIRC+KIRP)
KIRC	Kidney renal clear cell carcinoma
KIRP	Kidney renal papillary cell carcinoma
LAML	Acute Myeloid Leukemia
LGG	Brain Lower Grade Glioma
LIHC	Liver hepatocellular carcinoma
LUAD	Lung adenocarcinoma
LUSC	Lung squamous cell carcinoma
MESO	Mesothelioma
OV	Ovarian serous cystadenocarcinoma
PAAD	Pancreatic adenocarcinoma
PCPG	Pheochromocytoma and Paraganglioma
PRAD	Prostate adenocarcinoma
READ	Rectum adenocarcinoma
SARC	Sarcoma
SKCM	Skin Cutaneous Melanoma
STAD	Stomach adenocarcinoma
STES	Stomach and Esophageal carcinoma
TGCT	Testicular Germ Cell Tumors
THCA	Thyroid carcinoma
THYM	Thymoma
UCEC	Uterine Corpus Endometrial Carcinoma
UCS	Uterine Carcinosarcoma
UVM	Uveal Melanoma
RAB42	member of RAS oncogene family
OS	overall survival
DSS	disease-specific survival
DFI	disease-free interval
PFI	progression-free interval
PPI	PPI
GO	Gene Ontology
KEGG	Kyoto Encyclopedia of Genes and Genomes

BP	Biological process
CC	Cellular component
MF	Molecular function
GSEA	Gene set enrichment analysis
IL2	Interleukin-2
STAT5	signal transducers and activators of transcription 5
TNFA	tumor necrosis factor alpha
NFKB	Nuclear Factor kappa-B
PI3K	phosphatidylinositol-3-kinase
PD-L1	programmed death ligand 1
PD1	prephenate dehydratase 1
CTLA4	cytotoxic T-lymphocyte associated protein 4
TMB	tumor mutation burden
MSI	microsatellite instability
MSI-H	microsatellite instability-high
APC	APC regulator of WNT signaling pathway
TP53	tumor protein p53
KRAS	KRAS proto-oncogene
PTEN	phosphatase and tensin homolog
CTNNB1	catenin beta 1
H3K27ac	histone 3 lysine 27 acetylation
BRD4	bromodomain containing 4
TCGA	The Cancer Genome Atlas
GTEx	Genotype-Tissue Expression
CCLE	Cancer Cell Line Encyclopedia

TABLE S2 Correlation of RAB42 expression with IC50 of drugs.

Gene	Drug	Correlation	Pvalue
RAB42	PF-03758309	-0.511408047037293	0.0000408533542768594
RAB42	TYROTHRICIN	-0.481014306343894	0.000132323538783043
RAB42	geldanamycin analog	-0.462869469486491	0.000253671202496619
RAB42	Barasertib	-0.457778387749244	0.000302541648685769
RAB42	TAK-901	-0.427301152673435	0.000821730302376078
RAB42	Paclitaxel	-0.414744843543658	0.00120805305698054
RAB42	Volasertib	-0.410790383990264	0.00135980716448898
RAB42	Vinblastine	-0.404707482063116	0.001626770085215
RAB42	ARQ-621	-0.404167274269667	0.00165260840525535
RAB42	Eribulin mesilate	-0.401203921484764	0.00180099043162863
RAB42	PKI-587	-0.3994284060871	0.00189549668658586
RAB42	EMD-534085	-0.396078132531717	0.00208596557996328
RAB42	SGI-1027	-0.375377429667674	0.00368955422794006
RAB42	NMS-1286937	-0.369915106593752	0.00426319889798629
RAB42	Pipamperone	-0.368124258140446	0.00446768741679944
RAB42	BMS-387032	-0.365589542792897	0.00477188027234197
RAB42	AT-7519	-0.363791071576029	0.00499863334062476
RAB42	ONX-0914	-0.354277282089839	0.00636287091802706
RAB42	DOLASTATIN 10	-0.348893933204888	0.00727100516756416

RAB42	Actinomycin D	-0.345853294323162	0.00783237005926359
RAB42	Sepantronium bromide	-0.335879702338896	0.00994721917955311
RAB42	Des-fluoro-TAK-960	-0.335027358477202	0.0101489983531656
RAB42	TAK Plk inhibitor	-0.332388616564902	0.0107963186283264
RAB42	Vinorelbine	-0.329909222008758	0.0114367779477434
RAB42	GSK-461364	-0.325264200680427	0.0127252798228941
RAB42	SNX-5422	-0.30987188333679	0.0179266389945665
RAB42	Homoharringtonine	-0.30871731052872	0.0183810400731057
RAB42	Dinaciclib	-0.303877169791182	0.0203942113737487
RAB42	VINORELBINE	-0.301430936205484	0.0214809116030088
RAB42	Crizotinib	-0.295999551624129	0.0240700817316222
RAB42	TAK-960 analog	-0.295137473431892	0.0245043140008297
RAB42	SB-590885	-0.291428347102687	0.0264486925691044
RAB42	Depsipeptide	-0.290598227675576	0.0269011696272752
RAB42	Bafetinib	-0.284386445824753	0.030497720357092
RAB42	A-1210477	-0.279509650480056	0.0335953387246565
RAB42	MLN-2480	-0.279195626611842	0.0338034575032783
RAB42	TAK-632	-0.275259618578387	0.0365042539403081
RAB42	JNJ-47117096	-0.271459578272118	0.0392790991299163
RAB42	Carfilzomib	-0.271247228208099	0.0394391636727679
RAB42	ICG-001	-0.270595198317755	0.0399340183966344
RAB42	PF-4942847	-0.270159069369475	0.0402678678844733
RAB42	Litronesib	-0.267988645343624	0.041963711284088
RAB42	Danusertib	-0.265183599526917	0.0442420793570055
RAB42	BI-2536	-0.263789199064654	0.0454119028197045
RAB42	BGB-283	-0.261414574379898	0.0474623827826353
RAB42	VX-944	-0.260763786908769	0.0480373685279272
RAB42	AMG-900	-0.260271586372535	0.0484760103091654
RAB42	Tamoxifen	-0.259660724761104	0.0490249463454888
RAB42	Benzimate	-0.259610884313699	0.0490699573002912

TABLE S3 Docking score of three target drugs.

Name	Pose	Grid_Score	Grid_vdw_energy	Grid_es_energy	Internal_energy_repulsive
AT-7519	1	-57.760082	-55.330215	-2.429866	14.975024
BI-2536	1	-69.685188	-70.835358	1.150170	32.405167
BI-2536	2	-65.360077	-64.647903	-0.712177	13.895164
Dinaciclib	1	-63.034576	-57.326336	-5.708241	10.130095
Dinaciclib	2	-61.944305	-54.145233	-7.799074	9.552348

Table S4 RAB42-related lncRNAs

Gene Name	High expression in cancers	Positive correlation in cancers
AL031428.1	KIRC, STAD	ESCA, PCPG, LIHC
HAGLR	LIHC, CHOL, THCA, HNSC, ESCA,	CHOL, LAML, ACC, LIHC, ESCA
FGD5-AS1	CHOL	HNSC, CHOL, PRAD, LUSC, LIHC
CKMT2-AS1	LIHC	HNSC, THYM, BLCA
EPB41L4A-AS1	CHOL, KIRC	ACC, HNSC

AC021078.1	CHOL, KIRC, LIHC	LIHC, PAAD, LGG, THCA
ERICD	CHOL, LIHC, COAD, HNSC	HNSC, ESCA, LIHC, THYM, STAD
AL158206.1	THCA	LAML, UVM, ESCA, LIHC, THCA
H19	COAD, STAD	PAAD, LGG, THCA, LIHC, STAD
NEAT1	KIRC, PRAD, LIHC, KICH	LGG, TGCT, PAAD, UCEC, LIHC
MALAT1	COAD, KIRC, CHOL, LIHC, PRAD,	LIHC, ESCA, PAAD, PRAD
AC024940.6	CHOL, STAD, COAD, BLCA, KICH	LIHC, ESCA
HOTAIR	LUSC, STAD, LUAD, HNSC, COAD	LGG, DLBC, LIHC, LAML, PRAD
AL161772.1	CHOL, BLCA, LIHC, ESCA, COAD	UVM, LIHC, SARC, BRCA
AL137782.1	COAD	CHOL, THYM, THCA, PCPG, LIHC
AC015871.3	CHOL, LIHC,	ESCA, HNSC, LIHC, TGCT, CESC
AC087477.2	CHOL, KIRP	UVM, HNSC, LGG, PAAD, PRAD
ARHGAP27P1-BPTFP1-KPNA2P3	CHOL, KIRC, LIHC, KIRP, BLCA	KICH, CHOL, LIHC, UVM, THCA
SNHG16	COAD, CHOL	CHOL, ACC, LIHC, KIRC, PAAD
AC021092.1	CHOL, LUSC, LIHC, BLCA, LUAD	HNSC, LIHC, ESCA, LGG, CESC
AC243964.3	STAD, CHOL, LIHC	LAML, BLCA, BRCA, CESC, UCEC
AC005261.1	CHOL, LIHC, COAD	LIHC, LGG
NORAD	CHOL	UVM, LIHC, ESCA, PRAD, HNSC
XIST	KIRC	TGCT, LIHC
NPPA-AS1	KIRC, LIHC, STAD, HNSC, PRAD	KICH, LIHC, LGG, SARC, KIRP
MIR17HG	COAD, STAD, LIHC, KIRC, HNSC	ACC, ESCA, LAML, THCA, UCEC
AL049840.4	CHOL, LIHC, KIRC	UVM, LIHC, TGCT, LGG, THCA
AC125257.1	CHOL, STAD, ESCA, LIHC	UVM, KICH, HNSC, LIHC, TGCT
GAS5	CHOL, LIHC, KIRC, COAD, LUAD	ACC, KICH, LIHC
AL049543.1	CHOL, BLCA, STAD, LUSC, LIHC	THYM, LIHC, LAML, STAD, BLCA
HCP5	CHOL, HNSC, LIHC, KIRC, KICH	TGCT, UVM, LGG, PAAD, THCA
ZFAS1	CHOL, COAD, KIRC, LIHC, KIRP	ACC, KICH, LIHC, KIRC
MIRLET7BHG	CHOL, COAD, KIRC, LIHC, BLCA	UVM, LIHC, THCA
U62317.3	HNSC, KIRC, ESCA, CHOL, KIRP	KICH, UVM, LIHC, ACC, TGCT
LINC01184	CHOL	THYM, HNSC, TGCT, LIHC
EBLN3P	CHOL	PCPG, HNSC, PRAD, BLCA, LIHC
MIR497HG	KIRC	KICH, HNSC, ESCA, UVM, LUSC
AC092295.2	CHOL, LIHC	HNSC, PCPG, LUAD, LIHC, TGCT
AL356488.2	COAD, BRCA	UCS, ESCA, HNSC, BLCA, LUSC
AC234582.1	CHOL, LIHC	KICH, UVM, HNSC, PRAD, THCA
AC069281.2	CHOL, KICH, ESCA, KIRC	ESCA, HNSC, TGCT, LIHC
KCNQ1OT1	CHOL, LUAD, STAD, CHOL, KIRC	LIHC, ESCA, HNSC, LAML, PAAD
AC016876.2	KIRC, CHOL, KIRP	KICH, LIHC, KIRC, STAD, BRCA
AC073548.1	KIRC, CHOL, LIHC, HNSC, KICH	THYM, TGCT, LIHC, UCEC
AC010327.5	CHOL, LIHC	LIHC, HNSC, PRAD, THYM, TGCT
MIR663AHG	LIHC, STAD, HNSC, BRCA	KICH, CHOL, LIHC, THYM, UVM
